# Significance of thyroperoxidase and thyroglobulin antibodies in medically treated Graves’ disease

**DOI:** 10.1530/ETJ-23-0193

**Published:** 2023-11-30

**Authors:** Stefan Matei Constantinescu, Julien Hospel, Chantal Daumerie, Orsalia Alexopoulou, Dominique Maiter, Maria-Cristina Burlacu

**Affiliations:** 1 Department of Endocrinology and Nutrition, Cliniques Universitaires Saint-Luc, Université catholique de Louvain, Bruxelles, Belgium

**Keywords:** anti-thyroid antibodies, Graves’ disease, relapse, TED

## Abstract

**Background:**

Thyroperoxidase (TPOAb) and thyroglobulin (TgAb) antibodies are highly prevalent in Graves’ disease (GD), but their significance is controversial.

**Methods:**

We retrospectively analyzed TPOAb and TgAb levels and evolution in 136 patients with newly diagnosed GD between 2000 and 2022, treated with anti-thyroid drugs (ATD) in a block-and-replace (B+R) regimen for at least 12 months and followed up for at least 1 year after ATD discontinuation or until disease relapse.

**Results:**

At diagnosis, 98 out of 136 (72%) patients were TPOAb positive and 73 out of 136 (54%) patients were TgAb positive. The presence of TPOAb or TgAb antibodies at diagnosis was generally not related to GD presentation and did not influence the risk of relapse (*P* = 0.304 and *P* = 0.348, respectively). There was less TED (thyroid eye disease) in TgAb-positive patients than TgAb-negative patients at diagnosis (11 out of 73 (15.1%) versus 21 out of 63 (33.3%) *P* = 0.012). In contrast, the presence of TPOAb at diagnosis was not associated with TED (*P* = 0.354). The absence of TgAb at diagnosis (*P* = 0.05) and time to euthyroidism (*P* = 0.009), but not smoking or TRAb levels, were associated with TED in multivariate logistic regression. TPOAb and TgAb levels during treatment and after its discontinuation were not predictive of relapse, except for lower titers of TgAb at 18 months in patients who relapsed (*P* = 0.034).

**Conclusion:**

In GD patients treated with a first course of ATD in a B+R regimen we observed lower titers of TgAb at the end of treatment in patients who relapsed and a significant protection against TED in patients with positive TgAb at diagnosis, irrespectively of TPOAb.

## Introduction

Graves’ disease (GD) is the most common cause of hyperthyroidism worldwide, with a cumulative lifetime risk of 3% in women and 0.5% in men (1). The disease is characterized by the presence of thyrotropin receptor-stimulating antibodies (TRAb) that are responsible for the major manifestations of hyperthyroidism, i.e. goiter and orbitopathy (2). Other thyroid antibodies, namely, thyroperoxidase (TPOAb) and thyroglobulin (TgAb) antibodies, are also frequently elevated in GD, but contrary to TRAb, these antibodies might have a different pathogenic activity (3, 4, 5), and their significance in GD presentation and evolution is controversial. Determination of TPOAb or TgAb titer is currently not required for the diagnosis of GD.

Anti-thyroid drugs (ATD) have become the first-choice treatment for newly diagnosed Graves’ hyperthyroidism; however, this approach is hampered by a 50–55% relapse rate after an appropriate course of ATD (6). The presence of TED (thyroid eye disease), smoking, larger goiter size, severe hyperthyroidism, and high TRAb titers have been associated with a higher risk of disease relapse after ATD. However, the predictive value of each of these risk factors is too low for accurate assessment of GD relapse in an individual patient. Studies have shown that TPOAb and TgAb are very prevalent in GD patients (72–85% for TPOAb and 29–51% for TgAb) (7, 8, 9, 10) but have also led to conflicting results regarding the association of these antibodies with GD relapse (11, 12). Consequently, TPOAb and TgAb positivity was not considered in two meta-analyses of risk factors associated with relapse of GD treated with a first course of ATD (13, 14).

The present study aimed to investigate TPOAb and TgAb association with hyperthyroidism presentation and relapse in patients treated with ATD for a first episode of GD.

## Materials and methods

We retrospectively analyzed the medical files of consecutive nonpregnant adult patients treated for a first episode of GD at Cliniques Universitaires Saint-Luc, Brussels between 2000 and 2022. We included only patients with available TRAb, TPOAb, and TgAb measurements at diagnosis and at 6, 12, and 18 months or at treatment discontinuation if it occurred later. The patients were treated with ATD (thiamazole or, in case of treatment adverse events, propylthiouracil) in a block-and-replace regimen (B+R) for at least 12 months and followed up after ATD discontinuation for at least 1 year or until disease relapse. The block-and-replace regimen is commonly employed in our center for the medical treatment of GD. The medical treatment was discontinued when TRAb titer became normal. Relapse was defined as recurrent biochemical hyperthyroidism with elevation of serum TRAb and/or suggestive imaging, or the necessity to perform total thyroidectomy after a first uninterrupted course of treatment. The diagnosis of GD was made on the basis of the association of low TSH, elevated free FT4 and/or elevated FT3, imaging compatible with GD and the presence of TRAb. We also included 12 patients with undetectable TRAb at diagnosis in whom the clinical picture was compatible with GD, with no possible alternative diagnosis and ^99m^Tc scintigraphy showing diffuse thyroid uptake and/or thyroid ultrasound showing a hypervascularized gland. In 3 of these 12 patients, TRAb became positive during follow-up. The presence of TED was defined as one or more of the following eye findings: soft tissue changes (moderate or severe eyelid/conjunctival redness, moderate or severe eyelid/periorbital swelling), proptosis above the upper normal limit (Asians: 18 mm, Caucasians: 20 mm, Blacks: 22 mm), diplopia (intermittent, inconstant, or constant), and decreased visual acuity attributable to TED. In case of clinical suspicion, the presence of TED was confirmed by a dedicated ophthalmologist from our center. TED activity and severity was defined according to the EUGOGO classification (15).

TPOAb and/or TgAb positivity was defined as an antibody level superior to the upper normal limit of the assay. TRAb were measured by ELISA (Medizym, Medipan) between 2000 and 2016 and then by immunoassay (TRAK, Kryptor, Thermofisher) after 2016. TPOAb and TgAb, TSH, free T4 and T3 were measured by immunoassay with chemiluminescence using the Centaur system (Siemens) until 2006, then with DxI (Beckman Coulter) during 2006 and 2014, and finally by electrochemiluminescence on Cobas e602 (Roche Diagnostics) after 2014.

The study was approved by the local Ethics Committee (Comité d’Ethique Hospitalo-Facultaire des Cliniques Universitaires St-Luc, Université Catholique de Louvain, Brussels, Belgium).

### Statistical analysis

Statistical analyses were performed using the SPSS Statistics® software from IBM® (version 25.0). A *P*-value of less than 0.05 was considered significant. Continuous variables were described either as mean ± s.d. or median with 5th and 95th percentiles. Discrete variables were described using their frequency. Subgroup analyses were performed using Pearson’s *χ*² test for categorical unpaired variables. The Student’s *t*-test was used for comparing means of continuous unpaired variables when all subgroups were larger than 30 patients. Kruskal–Wallis’s test was used for comparing distributions of continuous variables between more than two subgroups with less than 30 patients.

## Results

### Population characteristics

Of the 327 initially selected patients, 161 patients were excluded because of missing TPOAb or TgAb during follow-up or insufficient follow-up, 17 patients for stopping medical treatment or receiving another treatment (radio-iodine or thyroidectomy) before having reached 12 months of treatment, and 13 for occurrence of pregnancy. The final analysis included 136 patients (Table 1) most of them (110/136, 81%) of Caucasian origin. The mean age at diagnosis was 41.7 ± 12.0 years, 105 out of 136 (77%) patients were women and 24 out of 136 (17%) patients were active smokers. TED was diagnosed in 32/136 (23.5%) patients: 11 patients developed moderate-to-severe TED requiring intravenous corticosteroids (of which 2 suffered from dysthyroid optic neuropathy) and 21 patients had mild TED. At diagnosis, a goiter was present in 86/136 (63%) of patients. In total, 116 (85%) patients were treated with thiamazole, 3 with propylthiouracil, and 17 with both drugs. Median duration of medical treatment was 18.0 (12.0–41.3) months, and 65 out of 136 (48%) patients were treated for more than 18 months. Euthyroidism was reached after a median of 7.0 (2.0-19.5) months. Median duration of follow-up was 44.0 (12.0–185.3) months and during this time, 74 out of 136 (54.4%) patients relapsed. Relapse occurred after a median of 9 (0–73.6) months after discontinuation of ATDs.

**Table 1:** Characteristics of the study population at baseline Results shown as mean ± s.d. or median (percentile 5–95).

Age (years)	41.7 ± 12.0
Gender: women/total	105/136 (77%)
Active smokers	24/136 (17%)
Medical treatment duration (months)	18 (12.0–41.3)
Follow-up (months)	44 (12.0–185.3)
TPOAb+ at diagnosis	98/136 (72%)
TgAb+ at diagnosis	73/136 (54%)
TED	32/136 (23%)
Relapse	74/136 (54%)

TgAb+, positive for anti-Tg antibodies; TPOAb+; positive for anti-TPO antibodies; TED, thyroid eye disease.

### TPOAb, TgAb, and GD presentation

At diagnosis, 98 out of 136 (72%) patients were TPOAb positive and 73 out of 136 (54%) patients were TgAb positive. There was no significant difference in terms of most patient characteristics (sex, smoking, goiter, TRAb titer, hyperthyroidism severity, time to euthyroidism, and duration of treatment) between TPOAb positive and TPOAb-negative patients at diagnosis or between TgAb-positive and TgAb-negative patients (Table 2). TPOAb-negative patients were older than TPOAb-positive patients at diagnosis (46.2 ± 11.7 years versus 40.0 ± 12.2 years, *P* = 0.008). There was less TED in patients who were TgAb positive at diagnosis than in patients without TgAb (11/73 vs 21/63, *P* = 0.012). In contrast, the presence of TPOAb was not associated with the presence of orbitopathy (*P* = 0.354). We performed unadjusted univariate logistic regression for the risk of TED and found time to euthyroidism and TgAb positivity to be significant predictors, and smoking and TRAb levels almost reached statistical significance (Table 3). TgAb and TPOAb levels, when considered as continuous variables, were not predictive of TED (Table 3). In a multivariate logistic regression adjusted for TRAb levels, delay to euthyroidism, and smoking, TgAb positivity resulted in an OR of 0.417 for the risk of TED (95% CI: 0.173–1.001, *P* = 0.050) (Table 3).

**Table 2:** Differences in patients’ characteristics with respect to TPOAb or TgAb positivity at diagnosis. Results shown as mean ± s.d. or median (percentile 5–95).

	TgAb			TPOAb		
	Positive	Negative	*P*	Positive	Negative	*P*
*n*	73	63		98	38	
Age	41.01 ± 12.28	42.54 ± 12.39	0.333	40.00 ± 12.16	46.16 ± 11.69	**0.008**
Sex, male/female	19/54	12/51	0.453	22/76	9/38	0.878
Smoking	12/73	12/63	0.691	18/98	6/38	0.723
Goiter	49/73	37/63	0.311	65/98	21/38	0.230
TRAb titer (×ULN)	5.92 ± 7.12	9.28 ± 17.17	0.131	8.15 ± 14.57	5.79 ± 6.84	0.339
T4 (×ULN)	2.15 ± 1.03	2.13 ± 1.06	0.923	2.19 ± 1.09	2.0 ± 0.91	0.344
Duration of treatment (months)	22.59 ± 10.20	24.68 ± 13.88	0.138	22.72 ± 11.77	23.79 ± 13.05	0.647
Time to euthyroidism (months)	7.44 ± 4.75	8.86 ± 6.13	0.131	8.12 ± 5.65	8.03 ± 5.02	0.927
TED	11/73	21/63	**0.012**	21/98	11/38	0.354
Relapse	37/73	37/63	0.348	56/98	18/38	0.513

Values in bold indicate statistical significance (*P* < 0.05).

TED, thyroid eye disease.

**Table 3:** Binary logistic regression of factors influencing the risk of TED. Values are odds ratio (95% CI).

	Univariate^a^	*P*	Multivariate^b^	*P*
Smoking	2.322 (0.902–5.974)	0.081	1.948 (0.684–5.548)	0.212
Delay to euthyroidism in months	1.132 (1.049–1.222)	**0.001**	1.112 (1.027–1.203)	**0.009**
TRAb levels/ULN	1.028 (0.997–1.060)	0.077	1.008 (0.979–1.038)	0.603
TgAb positivity (categorical)	0.355 (0.155–0.812)	**0.014**	0.417 (0.173–1.001)	**0.050**
TgAb levels/ULN (continuous)	0.974 (0.928–1.022)	0.288	–	–
TPOAb levels/ULN (continuous)	0.999 (0.994–1.004)	0.688	–	–

TED, thyroid eye disease. Values in bold indicate statistical significance (*P* < 0.05).

^a^Unadjusted; ^b^adjusted for smoking, delay to euthyroidism, TRAb levels/ULN, and TgAb positivity.

At diagnosis, TPOAb and TgAb were both positive in 64 out of 136 (47.1%) patients (group 1), 36 out of 136 (26.5%) patients had only TPOAb (group 2), 10 out of 136 (7.4%) patients had only TgAb (group 3), and neither antibody was positive in 26 out of 136 (19.1%) patients (group 4). There was no significant difference in terms of most patient characteristics (sex, smoking, goiter, TRAb titer, hyperthyroidism severity, treatment duration) between the four groups. There was a significant difference in terms of TED occurrence between the four groups, with less cases of TED in group 1 (*P* = 0.01) (Table 4).

**Table 4:** Presence of TED (thyroid eye disease) at diagnosis and relapse of Graves’ disease according to thyroid antibodies presence at diagnosis.

	TPOAb+ TgAb+	TPOAb+ TgAb−	TPOAb− TgAb+	TPOAb− TgAb−	*P*
Patients, *n*	64/136 (47%)	36/136 (26%)	10/136 (7%)	26/136 (19%)	
Relapse	31/64 (48%)	25/36 (69%)	5/10 (50%)	13/26 (50%)	0.214
TED	7/64 (11%)	14/36 (39%)	3/10 (30%)	8/26 (31%)	**0.01**

TgAb+/−: anti-Tg antibodies positive/negative; TPOAb +/−, anti-TPO antibodies positive/negative; TED, thyroid eye disease. Values in bold indicate statistical significance (*P* < 0.05).

### TPOAb, TgAb, and GD relapse

**Figure 1:**
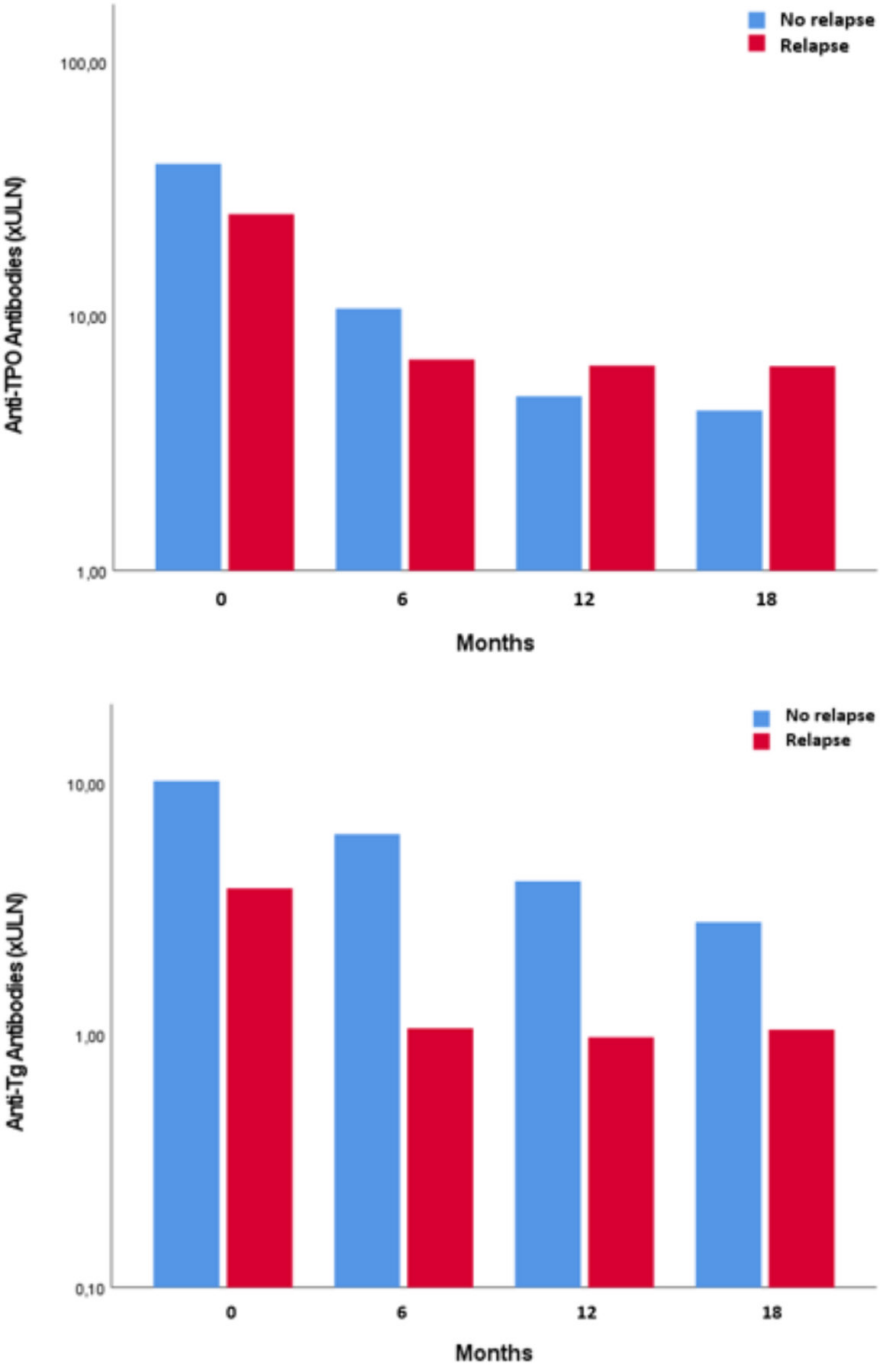
Evolution of thyroglobulin antibodies and thyroid peroxidase antibodies titers during the first-line medical treatment of Graves’ disease, in relapsing patients and in nonrelapsing patients. Note the logarithmic scale was used for the representation of antibody titers. ULN, upper limit of normal.

GD relapse was associated with higher titers of TRAb, both at diagnosis and at the end of ATD treatment (TRAb in UI/L, 39.8 ± 50.4 vs 16.5 ± 26.7 , *P* < 0.001 and TRAb in UI/l 0.7 ± 1.4 vs 6.8 ± 14.3, *P* < 0.001, respectively). TED was more prevalent in patients who relapsed than in patients who did not (24/74 (32%) vs 8/62 (13%), *P* = 0.007). Time to reach euthyroidism was longer in patients who relapsed (9.5 ± 6.2 vs 6.4 ± 3.8 months, *P* < 0.001). Sex, presence of a goiter, and smoking were not associated with relapse. Patients who relapsed were slightly younger than patients who did not (41.1 ± 12.3 vs 42.5 ± 12.4 years, *P* = 0.51). Also, 56 of the 98 (57%) TPOAb-positive patients and 37 of the 73 (51%) TgAb-positive patients relapsed. The presence of TPOAb or TgAb at diagnosis did not influence the risk of relapse (*P* = 0.304 and *P* = 0.348, respectively). There was no difference in terms of GD relapse between the four groups of antibodies at diagnosis, either (48%, 69%, 50%, and 50%, respectively, *P* = 0.214) (Table 4). The positivity of TPOAb and/or TgAb at treatment discontinuation or at the last visit before relapse was not predictive of relapse (data not shown). TPOAb titers decreased during treatment and were not significantly different between relapsing and nonrelapsing patients (Fig. 1). TgAb titers also decreased during treatment in all patients. We observed lower titers of TgAb during treatment in patients who relapsed (Fig. 1); however, this difference was not significant except at 18 months (*P* = 0.034).

In the relapsing patients, the evolution of TRAb, TPOAb, and TgAb during treatment was similar, with a decrease and nadir between 12 and 18 months, which was maintained at the last follow-up before recurrence, followed by elevation of antibody titers at the time of recurrence.

### Discussion

We show in this study that TPOAb and/or TgAb positivity at diagnosis was generally not associated with GD presentation or relapse after a first course of ATD treatment in a B+R regimen. Lower TgAb levels at the end of treatment were associated with an increased risk of relapse. Our data also indicate that the absence of TgAb at diagnosis was associated with a significant increase in TED risk.

We were able to reproduce previous findings such as a high proportion of TPOAb and TgAb positivity among GD patients, which is consistent with data from other published cohorts. We also observed a lower TPOAb positivity in older patients with GD (16). In a recent study in the general population, older age was associated with lower odds of TPOAb in the detectable and positive range (17). Similarly to our study, TPOAb-positive patients were younger than TPO negative patients in a Belgian cohort of GD addressed for radioiodine treatment, at a similar prevalence of TPOAb (69%) (18).

To our knowledge, there are very few studies about the association between the clinical presentation of GD and TPOAb and, let alone TgAb. In a very recent Japanese study, 442 patients with GD were divided in four groups according to TPOAb and TgAb positivity at diagnosis (11). The FT3-to-free thyroxine (FT4) (FT3/FT4) ratio was significantly higher and thyrotropin-stimulating hormone (TSH) receptor antibodies (TRAbs) were significantly lower in the TgAb+/TPOAb− group. Patients positive for TgAb developed GD with lower TRAb titers and underwent earlier remission than those negative for TgAbs. TPOAb-positive patients developed GD with high TRAb titers and needed a long time to achieve remission. We did not confirm this observation with respect to antibodies at diagnosis, but lower TgAb levels during treatment were associated with an increased risk of relapse.

Several small studies in populations with different ethnic background reported contradictory results with respect to baseline TPOAb and/or TgAb association with GD relapse. In an earlier Japanese study of 117 patients followed up for 30.6 months, patients with neither antibody before or during treatment were most likely to have a relapse of GD than those positive for both antibodies (11). Similarly, Indian patients with TPOAb- and TgAb-negative GD treated with methimazole for 18 months and followed up for a mean of 24.8 months had a higher risk of relapse than those who were positive for both antibodies (44% versus 11%) (19). Finally, a 2020 Australian study of 107 patients treated medically for GD found that the absence of TPOAb significantly increased the risk of relapse (OR: 2.21) (20). On the other hand, the presence of TPOAb or of both antibodies at diagnosis was not predictive of GD relapse in French, Korean, and Chinese studies (21, 22, 23). Moreover, a higher titer of TPOAb at diagnosis was predictive of long-term, but not short-term, GD relapse in a Croatian study (24).

In some of these studies, only the evolution of TPOAb and/or TgAb during treatment or after ATD withdrawal revealed a relationship with the GD relapse. TPOAb levels are known to decrease during GD treatment (25). Lesser decrease of TPOAb during ATD treatment or increased titer of TPOAb and TgAb after treatment withdrawal were found to correlate with TRAb persistence and higher GD relapse (26, 27). However, a study of 75 patients in France treated with a block-and-replace regimen for 18 months and followed up for 36 months found that TPOAb decreased during treatment and rise again to pretreatment levels after drug withdrawal, both in patients who relapsed and in those who did not, and their evolution was not predictive of disease relapse (21). We also observed that in relapsing patients, not only TPOAb but also TgAb decreases during treatment and then rises again. We did not observe a significant correlation between TPOAb evolution during ATD treatment or after treatment withdrawal and GD relapse. However, the TgAb titer during treatment was lower in patients who relapsed.

Thyroid peroxidase (TPO) is a thyroid enzyme involved in thyroid hormone synthesis through iodination of thyroglobulin (Tg). Both TPO and Tg, along with TSH receptor, are major thyroid autoantigens, but Tg has a higher ‘immunogenicity’ score than either TPO or TSH receptor (28). In GD, similar to Hashimoto thyroiditis (HT), the extent of lymphocytic infiltration is correlated with TPOAb and TgAb levels (29) and, for some authors, it could explain a positive correlation between the presence of these antibodies and higher likelihood of GD remission by the bias of evolution to chronic thyroiditis. However, the extent of intrathyroidal antibody production and action may not be well reflected by peripheral antibody measurements. Moreover, the significance of TgAb in GD remains controversial, as some reports indicate that TgAb do not manifest antibody-mediated cell cytotoxicity similar to that of TPOAb antibodies (30). TPO antibodies can fix complement and may have a directly pathogenic role in autoimmune thyroid diseases (5). Both HT and GD are autoimmune thyroid diseases, but their genetic causes, associated immune system alterations and effects on the thyroid gland are fundamentally different. Moreover, new work focusing on noncoding RNA and microbiota in patients with autoimmune thyroid disease, showed distinct patterns of noncoding RNA (31) and gut bacteria (32) in patients with GD, HT, and healthy controls.

In our population of GD, after adjusting for TRAb levels, smoking, and time to reach euthyroid state, the absence of TgAb at diagnosis was associated with an increased risk of TED. There are very few studies, especially in European populations, on TgAb either alone or in relation with TPOAb and their association with TED. In a 3-year prospective study of 100 patients from Singapore, both the levels of TgAb and TPOAb were lower and that of TRAb higher in TED patients, and the odds ratios for individual TED features ranged from 2.8 to 7.9 in the absence of TgAb (33). In a retrospective study of 108 TED patients treated with anti-inflammatory therapy, the NOSPECS score was negatively associated with TgAb (*r* = −0.27, *P* < 0.01) but not with TPOAb, while both showed no association with the CAS score (26). Very recently, TgAb negativity at diagnosis was linked to a higher risk of TED needing anti-inflammatory and immunosuppressive treatment in a large cohort of Japanese patients with newly diagnosed GD (hazard ratio: 2.98 (1.96–4.59), *P* < 0.0001) (34). If the protective effect of TgAb is confirmed in larger studies, clinicians could use TgAb level as a further risk factor for TED, in addition to smoking, the clinical activity score, TRAb levels, and duration of hyperthyroidism, recently published as the PREDIGO score for predicting TED in GD (35). Tg can be detected in the orbit of patients with TED (36, 37), and serum Tg levels have been correlated with TRAb and orbitopathy presence and severity (38). Although still unproven, some authors supported in the past the hypothesis that TgAb could bind orbital thyroglobulin and initiate or contribute to orbital inflammation. In this scenario, low TgAb in TED patients could be explained by their absorption in orbital tissues (39). This hypothesis was first formulated by Kriss *et al.*, who showed increased lymphatic drainage from the thyroid gland of GD patients toward the orbital cavities (40) and proved that Tg–TgAb complexes could bind to extraocular muscles (41). However, Tg–TgAb immune complexes have not been found in orbital tissues, and mice models immunized with Tg do not develop TED. This might be because TED is mainly a T-cell driven phenomenon and mice models might not be susceptible to TED (42).

In our study, TPOAb presence at diagnosis was not associated with TED. High TPOAb levels were more often associated with TED in pediatric patients, but studies in adults are more controversial and most of them did not find any association between TPOAb and the risk of TED (43). These differences might be explained by the heterogeneity of study populations and methodological differences in study design.

The strength of our study is the availability of both TgAb and TPOAb measurements and the analysis of both antibodies during disease evolution and not only at initial presentation. This is also one of the few studies to date on the association of TPOAb and TgAb and GD presentation.

Our study has several limitations. Our observations can only be applied to GD patients treated with ATD in a B+R regimen, a less commonly employed treatment, at least in Europe. Our cohort is limited in size and the number of orbitopathies relatively low compared to TED prevalence in European populations (44), possibly related to lower prevalence of active smoking in our population. However, the low prevalence of TED is similar to that seen in more recent reviews (45) with up-to-date ophthalmologic evaluation and low smoking prevalence.

Another limitation is the interassay variability of anti-TgAb assays, as reviewed recently (46). To counter this, we considered patients TgAb positive only if they had consistently elevated TgAbs measured in the same laboratory.

## Conclusion

In conclusion, in GD patients treated with a first course of ATD in a block-and-replace regimen, we observed lower titers of TgAb during treatment in patients who relapsed and a significant protection against TED in patients with positive TgAb at diagnosis irrespective of TPOAb titer.

## Declaration of interest

The authors declare that there is no conflict of interest that could be perceived as prejudicing the impartiality of the study reported.

## Funding

This work did not receive any specific grant from any funding agency in the public, commercial, or not-for-profit sector.

## Statement of ethics

This study protocol was reviewed and approved by the ethics committee of the Cliniques Universitaires Saint-Luc. Written informed consent was not required due to the retrospective nature of the study.

## Data availability statement

The data that support the findings of this study are not publicly available, as the data contains information that could compromise the privacy of research participants but are available from MCB upon reasonable request.

## Author contribution statement

SMC and JH wrote the first draft of the manuscript. JH collected the clinical data and DM performed all statistical analyses. CD, OA, and DM cared for patients and provided revisions for the manuscript. MCB led the clinical study. All authors reviewed and approved the final version of the manuscript.
